# Vitrification Effects on the Transcriptome of *in vivo*-Derived Porcine Morulae

**DOI:** 10.3389/fvets.2021.771996

**Published:** 2021-11-12

**Authors:** Cristina Cuello, Cristina A. Martinez, Josep M. Cambra, Alejandro González-Plaza, Inmaculada Parrilla, Heriberto Rodriguez-Martinez, Maria A. Gil, Emilio A. Martinez

**Affiliations:** ^1^Department of Medicine and Animal Surgery, Faculty of Veterinary Medicine, International Excellence Campus for Higher Education and Research “Campus Mare Nostrum,” Institute for Biomedical Research of Murcia (IMIB-Arrixaca), University of Murcia, Murcia, Spain; ^2^Department of Biomedical & Clinical Sciences (BKV), BKH/Obstetrics & Gynaecology, Faculty of Medicine and Health Sciences, Linköping University, Linköping, Sweden

**Keywords:** morula, vitrification, transcriptome, gene expression, pig, embryo

## Abstract

Despite the reported promising farrowing rates after non-surgical and surgical transfers of vitrified porcine morulae and blastocysts produced *in vivo* (range: 70–75%), the pregnancy loss is 5–15 fold higher with vitrified than with fresh embryos. The present study aimed to investigate whether vitrification affects the transcriptome of porcine morulae, using microarrays and RT-qPCR validation. Morulae were obtained surgically from weaned sows (*n* = 13) on day 6 (day 0 = estrus onset). A total of 60 morulae were vitrified (treatment group). After 1 week of storage, the vitrified morulae were warmed. Vitrified-warmed and non-vitrified fresh morulae (control; *n* = 40) were cultured for 24 h to assess embryo survival by stereomicroscopy after. A total of 30 vitrified/warmed embryos that were deemed viable and 30 fresh control embryos (three pools of 10 for each experimental group) were selected for microarray analysis. Gene expression was assessed with a GeneChip® Porcine Genome Array (Affymetrix). An ANOVA analysis p-unadjusted <0.05 and a fold change cut-off of ±1.5 were set to identify differentially expressed genes (DEGs). Data analysis and biological interpretation were performed using the Partek Genomic Suite 7.0 software. The survival rate of morulae after vitrification and warming (92.0 ± 8.3%) was similar to that of the control (100%). A total of 233 DEGs were identified in vitrified morulae (38 upregulated and 195 downregulated), compared to the control group. Nine pathways were significantly modified. Go-enrichment analysis revealed that DEGs were mainly related to the Biological Process functional group. Up-regulated DEGs were involved in glycosaminoglycan degradation, metabolic pathways and tryptophan metabolism KEGG pathways. The pathways related to the down-regulated DEGs were glycolysis/gluconeogenesis, protein export and fatty acid elongation. The disruption of metabolic pathways in morulae could be related to impaired embryo quality and developmental potential, despite the relatively high survival rates after warming observed *in vitro*. In conclusion, vitrification altered the gene expression pattern of porcine morulae produced *in vivo*, generating alterations in the transcriptome that may interfere with subsequent embryo development and pregnancy after embryo transfer.

## Introduction

Cryopreservation (1–3) of porcine embryos is the best tool for exchange and conservation of genetics and therefore has important applications for agriculture and biomedical research (4, 5). Cryopreservation of porcine embryos has proven difficult due to their high amount of lipids, which makes them extremely sensitive to cold (6). In the last decade, significant progress has been made in this technique thanks to the development of vitrification protocols [reviewed in (5, 7–10)]. Advances in vitrification devices and protocols, particularly the open pulled straw system (OPS) (11) using superfine OPS (SOPS) (12) straws, have allowed excellent viability *in vitro*, reaching >80% with vitrified morulae and >90% in blastocysts (1, 3, 13); moreover, after non-surgical (14, 15) and surgical (15, 16) transfer of vitrified morulae and blastocysts and farrowing rates ranging from 38.9 to 75% have been achieved. However, although there are few published reports, higher pregnancy loss has been observed in transfers with vitrified embryos (10–20%) than with fresh embryos (<2.5%) (17). The causes of this increased pregnancy failure are unknown. Nowadays, we know several factors that influence vitrification of porcine embryos, such as the embryo donor (3, 18), the type and concentration of cryoprotectants (1), or the developmental stage of the embryo (3, 19). Vitrification is also known to negatively affect porcine embryo quality by increasing apoptosis levels (13, 20) or affecting ultrastructure (13). Vitrification has been found to alter embryo the expression of genes in several species (21–26); however, the vitrification impact on mammalian embryo transcriptome is not well understood because general transcriptome experiments on vitrified-warmed embryos are very limited. Vitrification of *in vitro*-produced (IVP) bovine embryos caused overexpression of apoptosis-related genes (25) and repression of genes involved in cell differentiation, cell adhesion and metabolism of lipids (26). Vitrification has also been shown to modify the expression of genes related to the stress response (21) in both IVP and *in vivo*-derived bovine blastocysts. With respect to porcine species, most studies are limited to IVP blastocysts and are based on RT-qPCR studies, limiting research to a small number of genes. These studies have shown that vitrification modifies the expression of imprinted genes IGF2R and IGF2 (22), it also upregulates HSPA1A gene which plays an important role in stress regulation (23). It was also reported that vitrified IVP porcine blastocysts showed repression of POU5F1, which is involved in embryo implantation (23). Recently, the genes expression profile of vitrified-warmed porcine blastocysts produced *in vivo* was reported (27), this is the only report to date that includes wide transcriptome coverage of vitrified porcine embryos. In this study (27), the expression of 205 was significantly affected in the vitrified blastocysts compared to the fresh blastocysts, and the effect of vitrification and warming was modest in terms of the number of differentially expressed genes and fold changes. The altered genes in this study (27) were mainly involved cell cycle pathways, cellular senescence, gap junction, and signaling for TFGβ, p53, Fox, and MAPK. Although all of these studies are useful, information on the effects of vitrification on the porcine embryo transcriptome was limited to blastocyst stage embryos. Considering the stage-specific gene expression profiles between morulae and blastocysts, which is proper of preimplantation embryonic development (28), and that the morula is also a suitable stage for vitrification and embryo transfer (5), studies focusing on the embryonic developmental stage of morulae are needed. Better insight into the effects of vitrification on embryonic gene expression may help identify molecular lesions that may be associated with reduced embryo developmental capacity and more frequent pregnancy loss after transfer of vitrified embryos. This information would allow a better understanding of the adaptation process of embryos after vitrification and warming. These studies should be performed on *in vivo*-derived embryos, which are currently the only embryos suitable for commercial embryo transfer in this species. Therefore, the aim of this study was to use microarrays to investigate the effects of vitrification on the gene expression profile of porcine *in vivo* derived morulae.

## Materials and Methods

### Chemicals

All chemical products used in the experiments were purchased from Sigma-Aldrich Química S.A. (Madrid, Spain) unless otherwise stated.

### Ethics Statement

This study was conducted in accordance with Directive 2010/63/EU EEC for animal experiments. The Ethical Committee for animal experiments (research code: 486/2018) of the University of Murcia pre-evaluated and approved all the experiments presented in this manuscript.

### Animals, Estrus Detection and Artificial Insemination

Landrace x Large-White hybrid sows (2–6 parities) from a commercial farm (Agropor S. A., Murcia, Spain) were used as embryo donors. The donors were subject to proper management of the farm, they were fed twice daily with a commercial ration that met their nutritional needs, and water was offered *ad libitum*. Synchronization of estrus of the donor females was done by weaning. Donor sows were checked for estrus once daily, beginning 24 h after weaning, with a vasectomized mature boar, allowing muzzle-to-muzzle contact. Sows were considered to be in estrus, when they exhibited standing estrus reflex in the presence of the boar during manual dorsal pressing. Only donors in which the interval between weaning and estrus was 4–5 days were used in this study. The sows were inseminated by post cervical artificial insemination 6 and 24 h after the onset of estrus. Sperm doses (45 mL containing 1.5 × 10^9^ spermatozoa) were purchased from a commercial center. Sperm doses were derived from ejaculates of tested boars that were extended in Beltsville Thawing Solution extender [BTS; (29)] and stored at 17°C for 24 h.

### Embryo Recovery and Assessment

On day 6 of the estrous cycle, day 0 being considered the beginning of estrus, the embryos were collected. Embryo donors were sedated with azaperone (Stresnil®, Landegger Strasse, Austria; 2 mg/kg body weight, i.m.). Sodium thiopental (B.Braun VetCare SA, Barcelona, Spain; 7 mg/kg body weight, i.v.) was used to induce general anesthesia, which was maintained throughout surgery with 3–5% isoflurane gas (IsoFlo®, Madrid, Spain). A laparotomy was performed in the middle of the abdomen to expose the genital tract. The number of corpora lutea in the ovaries was counted to calculate the ovulation rate. Embryos were collected as previously described (30) by flushing the lumen of the tip of each uterine horn with 30 mL of modified Tyrode's lactate (TL)-HEPES-polyvinyl alcohol (31) [TL-HESPES-PVA; (15)]. After rinsing, all collected embryos were morphologically assessed using a stereomicroscope at 60× magnification to evaluate embryonic quality and developmental stage. Structures with only one cell and embryos with delayed development were classified as unfertilized oocytes and degenerated embryos, respectively. To select morulae with good or excellent morphology for the experiment, the guide International Embryo Transfer Society (32) was followed. Morulae with disrupted zona pellucida were excluded. Selected morulae were washed three times in TL-HEPES-PVA, placed in Eppendorf tubes containing 1.5 mL of this medium and transferred to the University of Murcia (Spain) within 2 h of collection in a transportable incubator set at 39°C.

### Vitrification and Warming of Morulae

Vitrification and warming were performed according to a previously described protocol (16). The base medium (BM) for both procedures was TL-HEPES-PVA and all media were maintained at 39°C. Morulae were vitrified within 4 h of collection. During equilibrations 4–6 morulae were processed. Morulae were washed twice at 39°C in BM and then equilibrated for 3 min in BM, which contained 7.5% (v/v) of dimethyl sulfoxide and ethylene glycol, for 3 min; they were then treated for 1 min with BM, which contained 16% (v/v) of dimethyl sulfoxide and ethylene glycol, and 0.4 M sucrose. After the final equilibration, embryos were localized in a 1.5 μL droplet and packed by capillary action into the narrower part of a superfine open-pulled straw (SOPS; Minitüb, Tiefenbach, Germany). Immediately, the straw containing the embryos was immersed in liquid nitrogen. Vitrified morulae were kept in a container with liquid nitrogen for 1 week. Subsequently, morulae were warmed using the one-step dilution method (2). To do this, the SOPS device containing the morulae was removed from the liquid nitrogen and immediately immersed in BM containing 0.13 M sucrose. Then, the recovered morulae were equilibrated in this medium for 5 min, the morulae were washed once in BM and cultured.

### *In vitro* Culture of Morulae and Embryo Survival Assessment

Vitrified-warmed morulae were cultured *in vitro* for 24 h. Culture was performed in NCSU-23 (33) medium supplemented with 0.4 mg/mL bovine serum albumin (BSA) and 10% (v/v) fetal calf serum under a paraffin oil cover (NidoilTM, Nidacon, Mölndal, Sweden). Culture plates were maintained in an incubator at 38.5°C, humidity to saturation (97%) and 5% CO_2_ in air. After culture, embryo development and morphology were assessed by stereomicroscopy to evaluate embryo survival. Those vitrified-warmed morulae that had developed to the blastocyst stage and showed good or excellent morphology with blastocoele, inner cell mas and trophoblast cells clearly differentiated, and an intact zona pellucida were considered viable. The same viability criteria were applied to the fresh control morulae. Survival rate was defined as the percentage of viable embryos out of the total number of embryos evaluated. Only embryos deemed viable after culture were used for transcriptome analysis. All the embryos analyzed were at the full blastocyst stage.

### Preparation of Samples and Microarray Hybridization

Sample preparation and microarray hybridization were performed as previously described (27). An RNeasy Micro Kit (P/N 74004; Qiagen Iberica, Madrid, Spain) was used to extract total RNA from the samples. The quantity and quality of the RNA obtained were analyzed using a Nanodrop 2000 (ThermoFisher Scientific, Madrid, Spain) and a Bioanalyzer 2100 (Agilent, Santa Clara, CA, USA). RIN values were higher than eight for all samples. ss-cDNA was then prepared using the GeneChip 3' IVT Pico Reagent kit (P/N 902790; Affymetrix, ThermoFisher Scientific, Madrid Spain) using 650 pg of RNA from each sample. The quantity and quality of the synthesized ds-cDNA was assessed using Nanodrop 2000 and Bioanalyzer 2100. The ds-DNA targets were purified, fragmented and terminally labeled. A total of 4.5 μg of the fragmented and biotinylated ds-DNA was incorporated into a hybridization mix from a GeneChip Hybridization, Wash and Stain Kit (P/N 90720; Affymetrix) according to the manufacturer's recommendations. The preparation was then hybridized to the GeneChip® Porcine Genome gen (P/N 900624; Affymetrix), which reads 20,201 genes, representing broad coverage of the Sus scrofa transcriptome. After chip scanning, microarray data were managed using the program Affymetrix Expression Command Console (Affymetrix). All the processed samples fulfill the criteria of quality.

### Microarray Data Analysis

Intensity data from each GeneChip® array were normalized using the robust multiarray mean (RMA) method (34), with average intensity values processed to match background cleanup. An individual intensity value for each sample was obtained by log_2_ transformation and quantile normalization of the raw values. Statistical analysis and biological interpretation of the microarray data were performed using the programs Partek Genomics Suite and Partek Pathways (Partek Incorporated, St. Louis, USA). Principal component analysis (PCA) was applied to determine the distribution of the analyzed data set. Data were statistically analyzed by applying a single factor ANOVA. An unadjusted *p*-value of < 0.05 was used as threshold for significance of differentially expressed genes (DEGs). Overrepresented Gene Ontology (GO) terms and pathways were examined using the Kyoto Encyclopedia of Genes and Genomes (KEGG) database as a reference. The ClueGo v2.0.3 application from the Cytoscape v3.0.0 software (35) was used to obtain pathway networks. The criterion for pathway grouping was a kappa score (≥0.4). Data are presented as means ± SD.

### Quantitative Real-Time PCR Analysis

RT-qPCR analysis was done using RNA extracted from the same samples than those used for microarray analysis. RNA was reverse transcribed to cDNA using a Maxima H Minus First Strand cDNA Synthesis Kit (Thermo Fisher Scientific) according to the manufacturer's instructions. Primers for RT-qPCR were designed using Primer ExpressTM software v3.0.1 (Applied Biosystems, Foster City, CA, USA). Genes for validation were selected based on their biological significance and/or high fold change values. The sequences of the primers are listed in Table 1. qPCRs analysis was performed using iTaqTM Universal SYBR Green Supermix in 10-μL volumes containing 500 nM of each primer set. All reactions were performed in a QuantStudioTM 5 Real-Time PCR system (Applied Biosystems). The PCR protocol included an initial step of 2 min at 50°C to activate uracil DNA glycosylase and 10 min at 95°C for initial denaturation, followed by 40 cycles of 15 s at 95°C and 1 min at 60°C. Melting curve analysis was used to evaluate the specificity of each PCR by detecting a single peak on the dissociation curve profile. Previous tests with additional samples were performed to calculate the efficiency of each primer pair using the equation E = 10(-1/slope). Relative mRNA levels were quantified by the Pfaffl method (36). Peptidylprolyl isomerase A (PPIA) and Actin Beta (ACTB) were used as reference genes for normalization of the data (37). Efficiency for each gene was calculated using the equation E = 10(-1/slope). Saphiro-Wilk's test was used to assess the normality of the RT-PCR data and distributions were parametric. Then, the data were analyzed by Student's *t*-test using the IBM SPSS 24.0 statistical software (IBM, Chicago, IL, USA). Levene's test was performed to determine the homogeneity of variance. A *p*-value of 0.05 was considered statistically significant.

**Table 1:** Sequences of the primers used for validation of the microarray by quantitative real-time PCR (RT-qPCR) analysis.

**Gene symbol**	**Accession number**	**Primer**		**Size**	**Efficiency**
*DECR1*	NM_001190232.2	Forward (5'−3')	GCAATTCAGTGTGATGTGAGG	155	92.85
		Reverse (5'−3')	ATGGTCTTCCAGGCATTAGG		
*WDR35*	XM_021087810.1	Forward (5'−3')	AAGACACGCAGCACAAACTG	185	85.46
		Reverse (5'−3')	AGAAGTCTGAATGGGTTCCTCA		
*PLEKHB1*	XM_005667095.3	Forward (5'−3')	GAATTGGTTCGCCCTGTG	173	94.32
		Reverse (5'−3')	GAATTGGTTCGCCCTGTG		
*GPC4*	XM_001925471.7	Forward (5'−3')	AACTCCGAGCTGTTCAAGGA	176	97.63
		Reverse (5'−3')	TACTTGCTCACGCATTCCAG		
*ACAT2*	XM_001928345.4	Forward (5'−3')	CTTCAATGGTGCTTTGTCCA	194	89.66
		Reverse (5'−3')	CCGGAACAGAGTAGGGGATT		
ALDOB	XM_021066854.1	Forward (5'−3')	GCAGAGGATCAAGGTGGAGA	175	97.87
		Reverse (5'−3')	CCCCTTTTCCTTGAGGATGT		
ACTB^*^	XM_021086047.1	Forward (5'−3')	GGACCTGACCGACTACCTCA	103	98.3
		Reverse (5'−3')	GCACAGCTTCTCCTTGATGTC		
PPIA^*^	XM_021078519.1	Forward (5'−3')	AGAAGTCTGAATGGGTTCCTCA	100	98.12
		Reverse (5'−3')	CCAACCACTCAGTCTTGGCA		

* *Housekeeping gene*.

### Experimental Design

In this experiment, embryos were collected from 13 weaned sows in three replicates. A total of 60 morulae were subjected to vitrification and warming, and then cultured *in vitro* for 24 h. Fresh morulae (*n* = 40) were also cultured *in vitro* for 24 h and used as control. After culture, embryo survival rates were assessed. Three pools of 10 viable vitrified-warmed embryos (each pool contained embryos from four different donors) were processed. Three pools of fresh embryos, from the same donors, were also prepared after culture. After culture and viability assessment, embryos were stored in RNAase-free Eppendorf tubes containing 5 μL phosphate-buffered saline (PBS) and stored at −80°C until microarray analysis. A total of six genes (three up-regulated genes and three down-regulated genes based on the microarray data; Table 1) were selected for validation of the microarray results. RT-qPCRs reactions were performed in triplicate, three technical and three biological replicates.

## Results

### Embryo Collection and Embryo Viability

The mean number of corpora lutea in donor sows ranged from 14 to 25 (mean of 20.8 ± 3.6). The embryo collection recovery rate was 92.2% and of the collected structures after flushing, 96.0% were morulae and blastocysts (*N* = 239), the remaining structures were unfertilized oocytes and/or degenerated embryos. In this study, 90 morulae were used and the rest of the embryos were used in other experiments. After 24 h of *in vitro* culture, the survival rate of vitrified-warmed morulae (92.0 ± 3.3%) was similar (n.s.) to that of fresh control morulae (100%).

### Transcriptome Profile of Vitrified-Warmed Morulae

**Figure 1:**
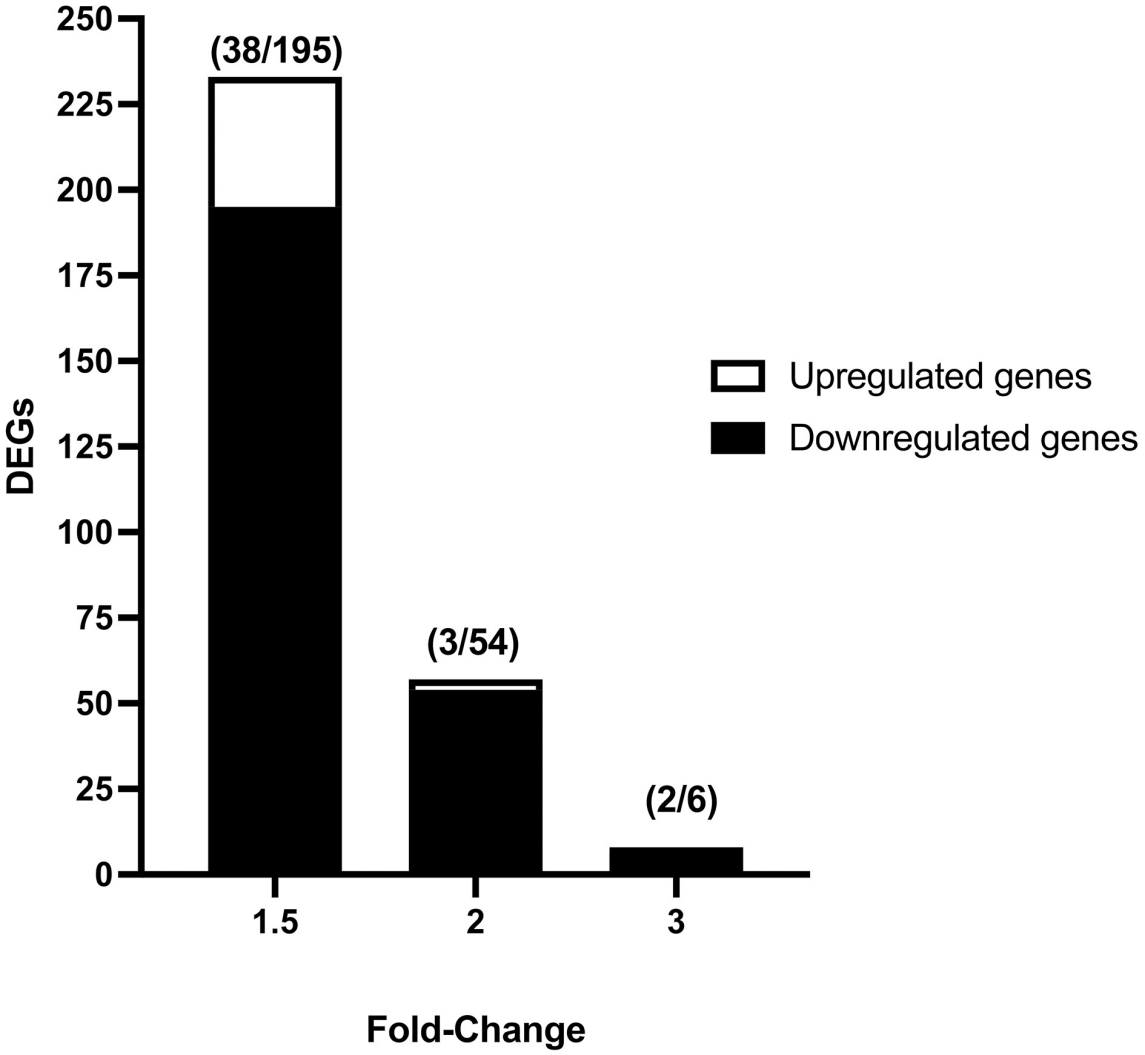
Number of differentially expressed genes (DEGs) in vitrified-warmed morulae compared to the fresh control embryos. DEGs were identified from transcriptome analysis using different fold-change values (1.5, 2, and 3) and an unadjusted *P* value of 0.05. Numbers in parentheses indicate the number of up- and downregulated DEGs.

**Figure 2:**
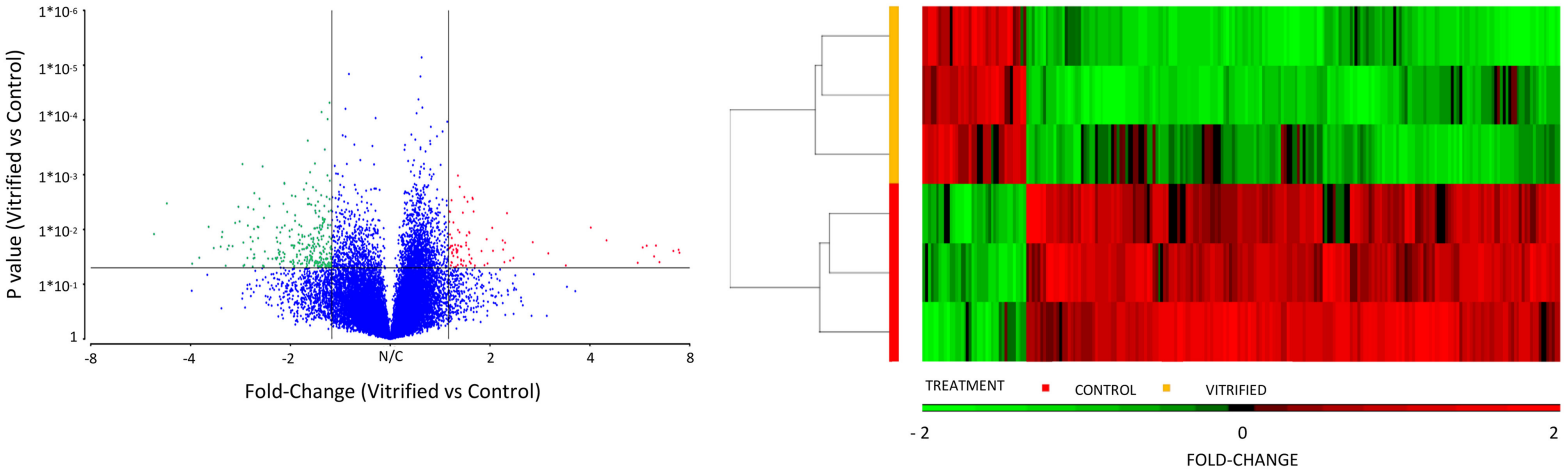
Volcano diagram of differential gene expression of vitrified morulae compared to control morulae. Differentially expressed genes (DEGs) were considered at a fold change cut-off value of 1.5 and a *P* value of 0.05. Red and green dots represent up- and downregulated DEGS, respectively. Unsupervised hierarchical clustering of genes that are significantly different in the analysis of gene expression patters of vitrified morulae compared with control morulae. The color scale under the heatmap represents the level of expression.

Vitrification and warming procedures significantly modified the transcriptome profile of porcine morulae. PCA analysis (Supplementary Figure 1) showed that treatment (vitrification or not) accounted for 71.0% of the variance. First, lists of differentially expressed genes (DEGs) were generated using an unadjusted *p*-value of 0.05 and different cutoff values for fold change; the resulting number of DEGs is shown in Figure 1. The DEGs list generated with the fold change −1.5 and 1.5 selection condition was used for successive analyses. Using this criterion, a total of 233 DEGs were observed in vitrified morulae in comparison with to control morulae (Supplementary Table 1). More specifically, 38 genes were upregulated while 195 were downregulated (Figure 1). The volcano plot in Figure 2 represents the DEGs detected in vitrified morulae. Unsupervised hierarchical clustering of the transcriptome samples showed that the vitrified samples could be clearly distinguished from the controls (Figure 2).

**Figure 3:**
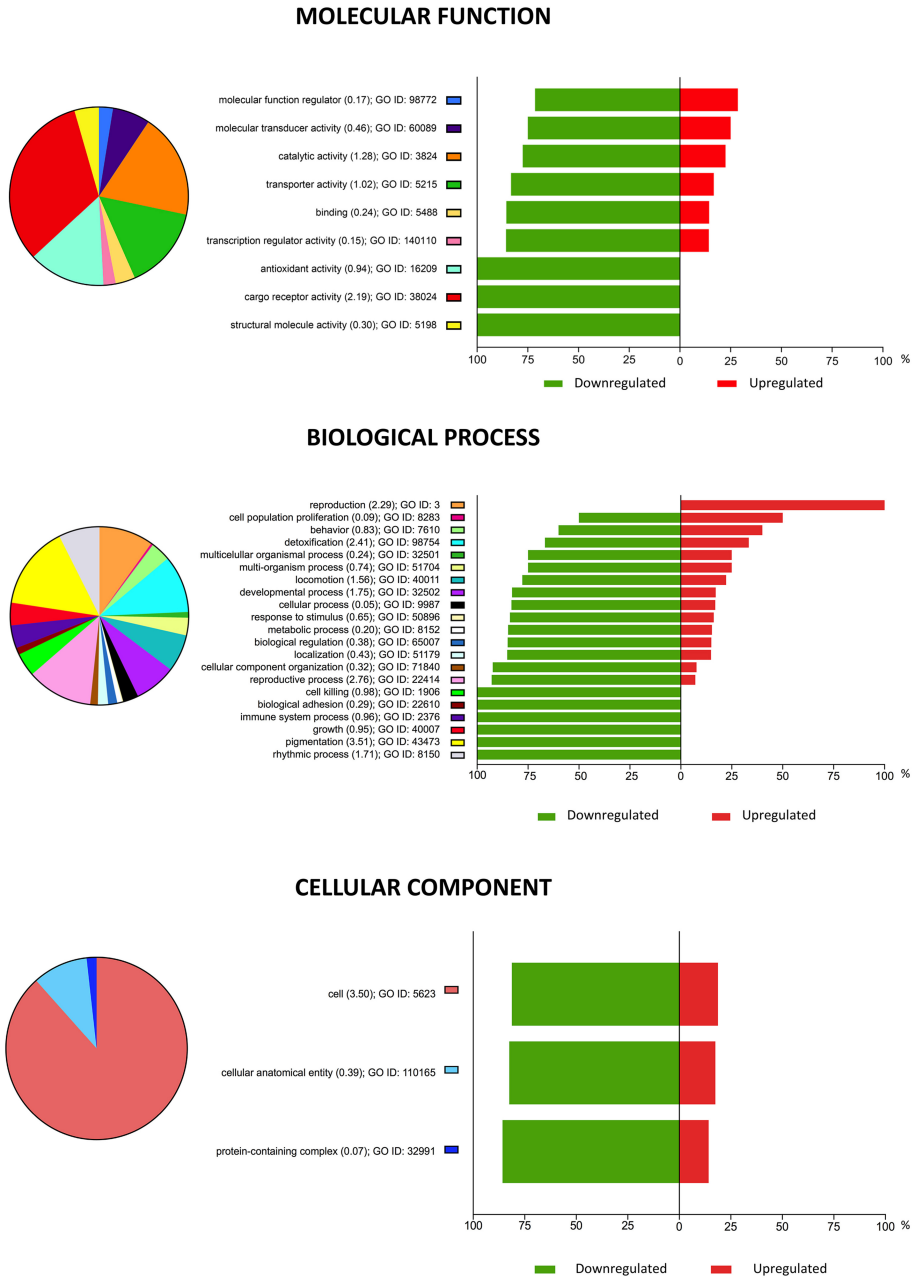
Pie chart representation of Gene Ontology (GO) for genes differentially expressed in vitrified morulae compared to control embryos, summarized by molecular function, biological processes, and cellular component. The plots show the proportion of differentially expressed genes (DEGs) within each functional group. Upregulated DEGs are shown in red and downregulated DEGs in green. Functional categories were analyzed based on the GO annotations of the KEGG classification system; the enrichment values of each GO term are shown in parentheses indicate.

Gene Ontology (GO) analysis was performed to categorize the major biological processes indicated by the DEGs. A total of 105 enriched GO terms with an enrichment *p* < 0.05 and an enrichment score ≥ 3 for vitrified morulae were identified, most of which were associated with biological processes. The top 10 most enriched GO terms are summarized in Table 2. Figure 3 depicts the categorization of DEGs in vitrified morulae in different functional categories related to biological process, molecular function, and cellular component. The biological function Response to zinc ion showed the highest enrichment score (10.2) and the 75% of the gene presented in this function were significantly modified in the vitrified embryos. Other biological functions with high enrichment scores were related to fatty-acyl-CoA synthesis and metabolism, the cellular response to fatty acids and lysosome organization.

**Table 2:** Top 10 most significant gene ontology (GO) terms for the differentially expressed genes in vitrified morulae.

**Biological Function**	**Type**	**Enrichment score**	**Enrichment *p*-value**	% genes in group that are present^*^
Response to zinc ion	BP	10.2	3.70E-05	75
Long-chain fatty-acyl-CoA biosynthetic process	BP	6.6	0.0013	66.7
Cellular response to fatty acid	BP	6.3	0.0018	25
3-oxo-arachidoyl (cerotoyl and lignoceronnyl)-CoA synthase activity	MF	5.9	0.0026	50
Very-long-chain 3-ketoacyl-CoA synthase activity	MF	5.9	0.0026	50
Long-chain fatty-acyl-CoA metabolic process	BP	5.9	0.0026	50
Fatty-acyl-CoA biosynthetic process	BP	5.9	0.0026	50
Rab guanyl-nucleotide exchange factor activity	MF	5.9	0.0026	50
Response to fatty acid	BP	5.8	0.0029	21.4
Lysosome organization	BP	5.3	0.0051	17.7

*BP, biological process; MF, molecular function.*

* *% of genes in group that are present in the differentially expressed genes list (vitrified vs control morulae)*.

### KEGG Pathways Enrichment Analysis of DEGs in Vitrified Morulae

Pathway enrichment analysis for the DEGs in vitrified morulae compared to control morulae identified 10 enriched KEGG pathways for upregulated (Table 3). Enriched pathways for upregulated genes were glycosaminoglycan degradation, tryptophan metabolism, metabolic pathways, synthesis and degradation of ketone bodies, glycosphingolipid biosynthesis, other glycan degradation, terpenoid backbone biosynthesis, butanoate metabolism and fatty acid elongation. The percentage of genes altered in these pathways ranged from 0.7 to 11.1%. The most enriched pathway was glycosaminoglycan degradation (7.6 enrichment score), the other nine enriched pathways showed enrichment values from 3.1 to 5.9. The upregulated DEGs in these pathways HEXA, SPAM1, ACAT2, HAAO, MAN1C1, PIP5K1A, PYGM and ELOVl.

**Table 3:** Enrichment analysis of pathways for up-regulated differentially expressed genes in vitrified morulae.

**Pathway name**	**Pathway ID**	**Enrichment score**	**Enrichment *p*-value**	**% genes of DEGs in pathway**	**Gene list**
Glycosaminoglycan degradation	kegg_pathway_48	7.6	0.0005	11.1	*HEXA, SPAM1*
Tryptophan metabolism	kegg_pathway_31	5.9	0.0029	4.7	*ACAT2, HAAO*
Metabolic pathways	kegg_pathway_87	5.5	0.0039	0.7	*ACAT2, HAAO, HEXA, MAN1C1, PIP5K1A, PYGM, SPAM1*
Synthesis and degradation of ketone bodies	kegg_pathway_11	4.1	0.0168	11.1	*ACAT2*
Glycosphingolipid biosynthesis-ganglio series	kegg_pathway_63	3.6	0.0279	6.7	*HEXA*
Glycosphingolipid biosynthesis-globo and isoglobo series	kegg_pathway_62	3.5	0.0298	6.3	*HEXA*
Other glycan degradation	kegg_pathway_42	3.4	0.0334	5.6	*HEXA*
Terpenoid backbone biosynthesis	kegg_pathway_79	3.3	0.0389	4.8	*ACAT2*
Butanoate metabolism	kegg_pathway_67	3.1	0.0443	4.2	*ACAT2*
Fatty acid elongation	kegg_pathway_9	3.1	0.0461	4.0	*ELOVL1*

Seven KEGG pathways were significantly enriched for downregulated DEGs (Table 4). The most enriched pathways related to downregulated DEGs were glycolysis-gluconeogenesis and protein export (4.3 and 4.2 enrichment score, respectively). Other pathways enriched for downregulated DEGs were fatty acid elongation, spliceosome, fructose and mannose metabolism, Wnt signaling pathway and endocytosis. The genes altered in these pathways were AKR1A1, ALDH1B1, ALDOB, SPCS3, and SRP72. The percentage of modified genes in the pathways enriched for downregulated genes ranged from 0.6 to 11.1%. Other genes altered in these pathways, in addition to those previously described, were ELOVL7, HACD4, HSPA2, RBMX, SNRNP40, SRSF6, FPGT, GPC4, LRP6, MAPK8, PPP3CB, ARAP2, ASP1, PARD6G and TFRC.

**Table 4:** Enrichment analysis of pathways for down-regulated differentially expressed genes in vitrified morulae.

**Pathway name**	**Pathway ID**	**Enrichment score**	**Enrichment *p*-value**	**% genes of DEGs in pathway**	**Gene list**
Glycolysis/Gluconeogenesis	kegg_pathway_1	4.3	0.0141	11.1	*AKR1A1, ALDH1B1, ALDOB*
Protein export	kegg_pathway_107	4.2	0.0150	4.7	*SPCS3, SRP72*
Fatty acid elongation	kegg_pathway_9	4.0	0.0176	0.6	*ELOVL7, HACD4*
Spliceosome	kegg_pathway_105	4.0	0.0181	11.1	*HSPA2, RBMX, SNRNP40, SRSF6*
Fructose and mannose metabolism	kegg_pathway_5	3.6	0.0281	6.7	*ALDOB, FPGT*
Wnt signaling pathway	kegg_pathway_158	3.6	0.0282	6.3	*GPC4, LRP6, MAPK8, PPP3CB*
Endocytosis	kegg_pathway_142	3.1	0.0444	5.6	*ARAP2, ASP1, HSPA2, PARD6G, TFRC*

**Figure 4:**
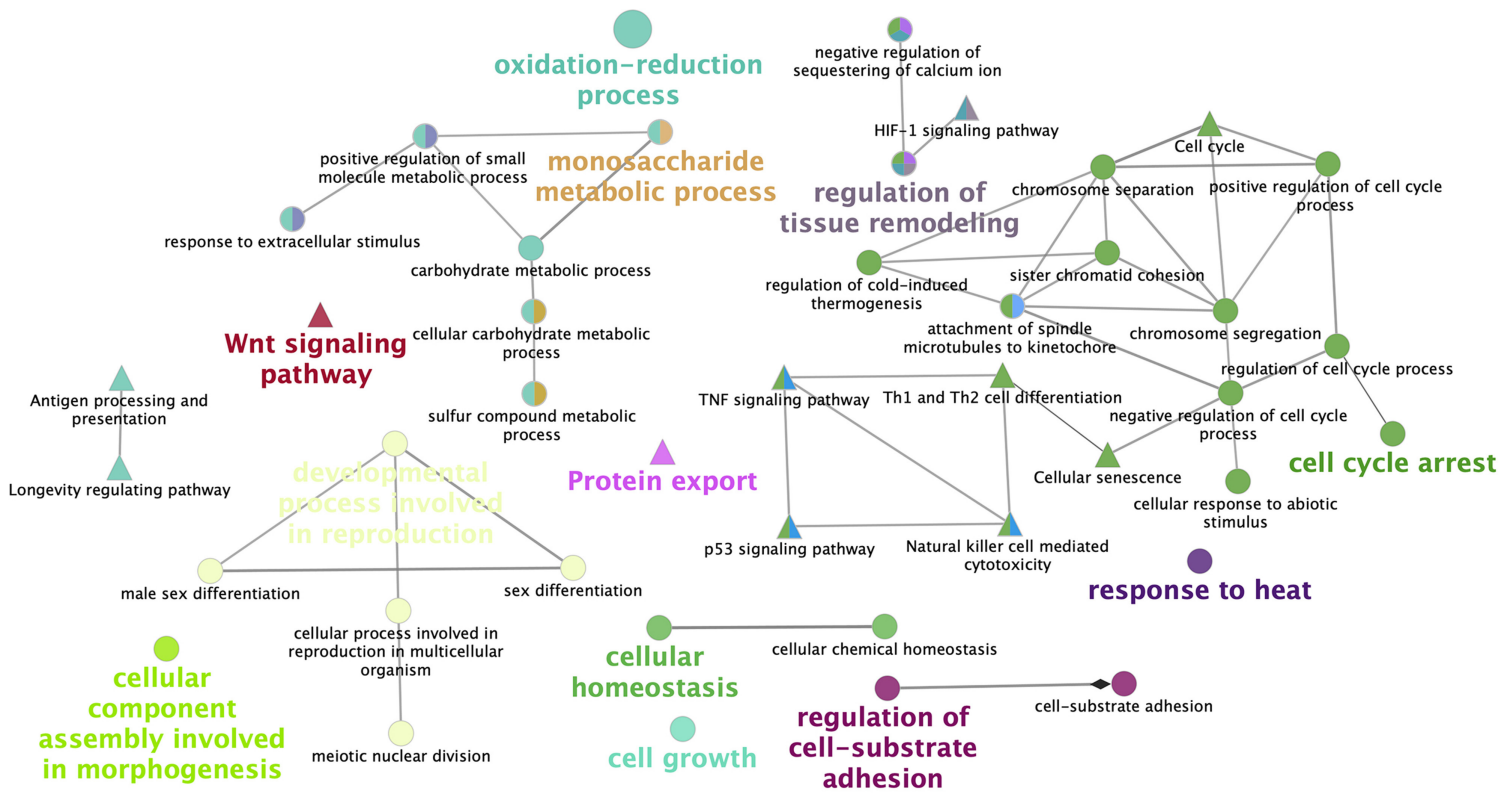
Network representation of enriched pathways obtained from the differentially expressed genes in vitrified morulae. ClueGo v2.0.3 (Cytoscape v3.0.0 software) shows the interaction of functionally grouped gene clusters. Nodes in the same cluster have the same color and the size of the node represents the significance of the enrichment. Groups are labeled attending of common processes within the cluster. Edges indicate interactions between functional groups.

Figure 4 represents the KEGG pathway networks analyzed with Cytoscape for vitrified morulae.

### Microarray Results Validation

**Figure 5:**
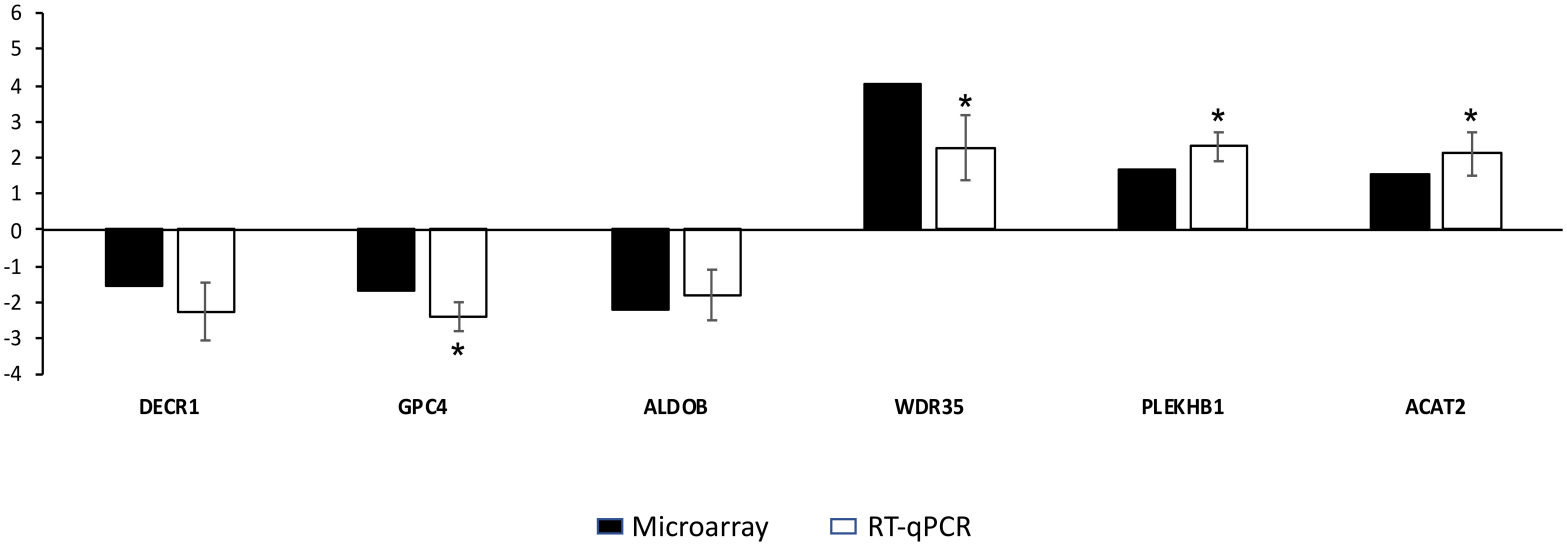
Results of the quantitative real-time PCR (RT-qPCR) used to validate microarray results. The y-axis indicates the fold change of each differentially expressed gene in vitrified morulae compared to control morulae. Asterisks represent significant differences between groups as determined by RT-qPCR analysis. Bars represent mean standard errors (SEM).

Microarray data were validated by RT-qPCR analysis. The six genes validated by RT-qPCR showed an expression trend similar to that obtained in the microarrays (Figure 5). The RT-qPCR validation showed that the mRNA levels for WDR25, PLEKHB1 and ACAT2 were significantly (*p* < 0.05) upregulated. The expression of GPC4 was significantly (*p* < 0.05) downregulated. Although the expression levels for DECR1 and ALDOB were similar for, the expression of these genes was coherent with the microarray results.

## Discussion

To our knowledge, this study represents the first description of the impact of vitrification and warming on the gene expression pattern of porcine morulae obtained *in vivo*. This study provides new information on the consequences of this technology on embryo quality and developmental competence. This knowledge may be important not only in swine but also in other mammals. According to the results of this study, the effect of vitrification on embryos at the morula stage in terms of the number of DEGs (233 DEGs) was similar to that previously reported for blastocysts (205 DEGs) (27). Although the number of DEGs and fold change values were similar to those previously described for blastocysts (27), vitrified morulae and blastocysts (27) showed very different transcriptome profiles, with only 12 (three upregulated and eight downregulated) DEGs as common features. This is not surprising considering that stage-specific (38–41) gene expression between morulae and blastocysts is characteristic of preimplantation embryonic development (28).

The enrichment analysis of the GO terms in vitrified morulae showed that, with the exception of the biological process “reproduction,” all the disturbed GO biological processes, GO cellular components and GO molecular functions were mainly suppressed. The most enriched GO terms in the morulae were related to fatty acid and acyl-CoA metabolism. It is well known that both fatty acids and acyl-CoA are metabolic switches associated with pluripotency and are of great importance in embryonic development (42, 43). Moreover, acyl-CoA controls crucial cellular processes such as mitosis, autophagy and energy metabolism, and this control is either direct or is mediated by epigenetic regulation of gene expression (44, 45). On the other hand, fatty acids are also key factors associated with metabolism, cell signaling, oxidative stress and gene expression in preimplantation embryos (46, 47). Downregulation of genes in these two functional groups could be detrimental to the development of vitrified morulae. It may be interesting to investigate whether supplementation of the vitrification-warming medium with acyl-CoA and/or fatty acids can offset these potential negative effects.

The enrichment analysis of KEGG pathways related to the up- and downregulated genes of vitrified morulae showed that the effects of vitrification were modest with regard to the number of modified pathways and the percentage of genes within each pathway that shows different expression (range 0.7–11.1%). However, we should pay special attention to the altered pathways that are important for embryonic development. Analysis of KEGG pathway based on upregulated DEGs in vitrified morulae, revealed that nine pathways were enriched. Genes upregulated in these pathways (HEXA, SPAM1, ACAT2, HAAO, MAN1C1, PIP5K1A, PYGM, and ELOVL1) have not previously been described as directly linked to embryonic development. The enrichment of the KEGG Pathway Metabolic pathways, which has also been described in vitrified *in vivo*-derived bovine blastocysts (48, 49), could be a sign of low embryo quality. Activation of this pathway and other metabolism-related pathways such as tryptophan metabolism and butanoate metabolism, may have negative consequences on the development and implantation of vitrified morulae according to the “quiet embryo” hypothesis, which affirms that survival of the preimplantation embryo is associated with relatively “quiet” metabolism (50–52).

ACAT2 is involved in the enriched pathways tryptophan metabolism, degradation of ketone bodies, terpenoid backbone biosynthesis and butanoate metabolism and metabolic pathways. The product of this gene is the enzyme thiolase II, also known as acetyl-CoA acetyltransferase II (53), which is involved in lipid metabolism. This enzyme converts two molecules of acetyl-CoA to acetoacetyl-CoA in the mevalonate (MVA) pathway in eukaryotes, which is the first step of isoprenoid production (54). It has been shown that thiolase II is a conserved enzyme that catalyzes the rate-limiting step in isoprenoid synthesis during the abiotic stress response (55) and therefore plays a fundamental role in the reduction of oxidative stress generated by abiotic factors such as temperature and dehydration (55). Overexpression of ACAT2 in vitrified morulae could represent a response to oxidative stress induced by vitrification (56). Along these lines, overexpression of this gene has been described in bovine blastocysts generated by nuclear transfer compared with control embryos generated *in vitro* (57). Although the effect of vitrification on porcine embryos produced *in vivo* has not been studied in detail, the addition of antioxidants has been shown to enhance the vitrificability of *in vitro* produced porcine blastocysts (56, 58, 59). Therefore, the effects of vitrification on the redox balance of embryos produced *in vivo* should be investigated, as well as the possible beneficial effects of the addition of antioxidants to the vitrification and warming media on these embryos.

HEXA and SPAM1 are upregulated DEGs common to the metabolic and glycosaminoglycan degradation pathways. HEXA gene encodes the instructions for the synthesis of a portion (alpha subunit) of beta-hexosaminidase A. This enzyme, in combination with cofactor GM2, catalyzes the degradation of ganglioside GM2 and other molecules containing terminal N-acetylhexosamines (53). SPAM1 encodes the enzyme hyaluronidase, this protein degrades hyaluronic acid, which is the major structural proteoglycan of the extracellular matrix and basement membrane (53). Dysfunctions in the Glycosaminoglycan degradation have been related to neuronal diseases in humans (60). In relation to embryonic and fetal development, glycosaminoglycans play an important role in cell growth, differentiation, morphogenesis, and cell migration (61, 62). The enrichment of the glycosaminoglycan degradation pathway may impair these functions and thus affect embryonic development.

Cell proliferation in vitrified morulae could also be affected by transcriptional changes in the glycolysis/gluconeogenesis and fructose and mannose metabolism pathways, which appeared enriched for downregulated DEGs. Prior to gastrulation, dividing cells favor glycolysis for efficient cell proliferation (63). During this period, the expression of aldolase genes, including ALDOB, which is repressed in vitrified morulae, becomes essential for the regulation of glucose metabolism (64). Furthermore, ALDOB promotes proliferation independent of its glycolytic role, due to its effects on the cell cytoskeleton (65).

Among the seven enriched pathways for downregulated genes in vitrified morulae, it is remarkably the Wnt signaling pathway that plays a fundamental role in embryonic development (50–52). The Wnt signaling pathway regulates cell proliferation and differentiation in mammalian embryos (66, 67). Prominent among the genes repressed in this pathway is GPC4, which encodes Glypican 4, a glycosylphosphatidylinositol-anchored heparan sulfate proteoglycan that modulates signaling by growth factors and Wnts. GPC4 has multiple roles during embryonic development (38, 68, 69). It has been reported that GPC4 facilitates ligand/receptor interactions in various developing tissues in the mouse embryo, thus the expression level of GPC4 appears to be functionally important (69), and therefore suppression of this gene could impair the developmental potential of vitrified morulae.

The fatty acid elongation pathway was found to be enriched in both upregulated and downregulated DEGs in this study. ELOVL1 was overexpressed in vitrified embryos, whereas ELOVL7 and HACD4 were suppressed in vitrified embryos. It is known that long-chain acyl CoA synthetases (ACSLs) and elongation of very long fatty acids enzymes (ELOVLs) are involved in membrane lipid metabolic pathways (70); and that the levels of mRNA transcripts related to fatty acid elongation are related to membrane chemical composition, as these changes are physiological during embryogenesis (71). However, the differential gene expression patterns observed in this study may be related to the embryo response to vitrification. In this regard, it has been described previously that vitrification alters the membrane lipid profile of bovine embryos produced *in vitro* (72). These authors have described how membranes remodel after vitrification and subsequent 24 h culture. This process reflects a repair response of embryo membranes to membrane damage caused by vitrification and warming (72). Porcine embryos, which like bovine embryos produced *in vitro* (73, 74) have high lipid content (6), may be more sensitive to these changes in long-chain fatty acid pathways caused by vitrification. In this sense, it would be interesting to investigate whether the long-chain fatty acid profiles could be used as potential biomarkers for embryo survival and quality after vitrification, as has been proposed for phosphatidylcholine and sphingomyelin in cryopreserved bovine blastocysts produced *in vivo* and in *vitro* (75).

Normalizing the expression patterns of vitrified morulae could improve the developmental potential of these embryos. Further investigations are required to discover the biological consequences of the effects of vitrification on the transcriptome of vitrified morulae and the strategies to minimize the negative effects.

## Conclusions

These results here presented demonstrate that vitrification alters the gene expression profile of *in vivo*-derived porcine morulae. The effect of vitrification on morulae consisted largely of suppression of gene expression, with the exception of metabolism-related pathways. More investigation is needed to elucidate the biological impact and significance of the GO terms and pathways altered by vitrification and to develop screening methods for warmed embryos that go beyond the apparently suboptimal morphological assessment currently in use.

## Data Availability Statement

The datasets presented in this study can be found in online repositories. The names of the repository/repositories and accession number(s) can be found at: Array Express, accession no: E-MTAB-11017.

## Ethics Statement

The animal study was reviewed and approved by the experiments were pre-evaluated and approved at the University of Murcia (Spain) by the Ethical Committee for animal experiments (research code: 486/2018). Written informed consent was obtained from the owners for the participation of their animals in this study.

## Author Contributions

Conceptualization: CC, CAM, MAG, HR-M, and EAM. Methodology: CC, CAM, JMC, IP, MAG, and EAM. Software: CC, CAM, and EAM. Validation: EAM and CC. Data curation: EAM. Writing-original draft preparation: CC, CAM, and EAM. Writing-review and editing: JMC, MAG, and HR-M. Visualization: CC. Supervision: CC, EAM, and HR-M. Project administration: CC and EAM. Funding acquisition: CC, MAG, and EAM. All authors have read and agreed to the published version of the manuscript.

## Funding

This research was funded by the Fundacion Seneca (19892/GERM/15), Murcia, Spain; the MCIN/AEI/10.13039/501100011033/“ERDF a way of makin Europe” (RTI2018-093525-B-I00), Madrid, Spain; the European Union's Horizon 2020 research and innovation program under the MSCA (grant agreement No 891663); and the Swedish Research Council FORMAS (Projects 2017-00946 and 2019-00288), Stockholm, Sweden.

## Conflict of Interest

The authors declare that the research was conducted in the absence of any commercial or financial relationships that could be construed as a potential conflict of interest.

## Publisher's Note

All claims expressed in this article are solely those of the authors and do not necessarily represent those of their affiliated organizations, or those of the publisher, the editors and the reviewers. Any product that may be evaluated in this article, or claim that may be made by its manufacturer, is not guaranteed or endorsed by the publisher.

## Supplementary Material

Supplementary Table 1 List of differentially expressed genes (DEGs) in vitrified-warmed morulae compared to the fresh control embryos. DEGs were identified from transcriptome analysis using different fold-change value of 1.5 and an unadjusted *P*-value of 0.05.

Supplementary Figure 1 Principal component analysis (PCA) of the microarray-based gene expression profiles of vitrified (red; *n* = 3) and control (blue; *n* = 3) morulae. Each data sphere represents a microarray-A three principal component is shown.
