# Supplementary figures and images for: Vitrification Effects on the Transcriptome of in vivo-Derived Porcine Morulae
Source: Front Vet Sci. 2021 Nov 12;8:771996. doi: 10.3389/fvets.2021.771996 (PMC8633305; doi:10.3389/fvets.2021.771996)

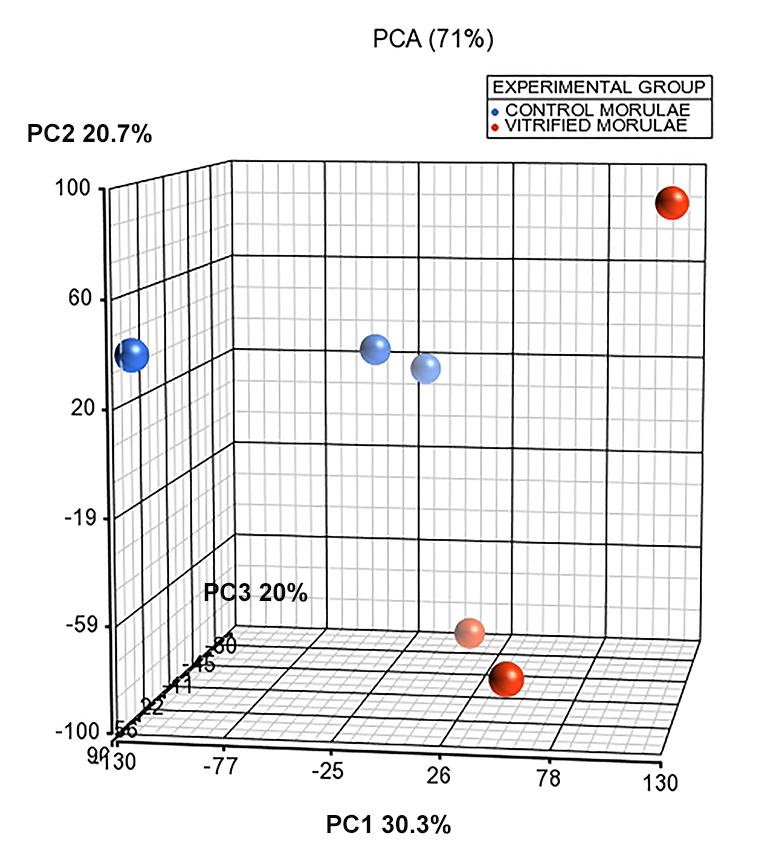

Supplement: Supplementary Figure 1 — Principal component analysis (PCA) of the microarray-based gene expression profiles of vitrified (red; n = 3) and control (blue; n = 3) morulae. Each data sphere represents a microarray-A three principal component is shown. [file Image_1.JPEG]
